# Flexural performance of RC beams strengthened with CFF and SCCFL sheets under cyclic loading

**DOI:** 10.1038/s41598-026-35884-w

**Published:** 2026-01-28

**Authors:** V. S. Sujitha, A. Gautham Sriram, S. Raja, Maher Ali Rusho, Simon Yishak

**Affiliations:** 1 Center for Advanced Multidisciplinary Research and Innovation, Chennai Institute of Technology, Chennai, Tamil Nadu 600069 India; 2 Lockheed Matin Engineering Management, University of Colorado, Boulder, CO 80308 USA; 3 College of Engineering and Agro-Industrial Technology, Sawla Campus, Arba Minch University, Arba Minch, Ethiopia

**Keywords:** Concrete, Flexure, Fatigue, Bond, Strengthening, Loading, Carbon, Laminate, Fabric, Engineering, Civil engineering

## Abstract

Carbon fibre reinforced polymer (CFRP) sheets have a high strength to weight ratio and corrosion resistance, and consequently they have attracted much attention for flexurally strengthening deteriorated reinforced concrete (RC) beams. The study experimentally evaluated the flexural performance of RC beams strengthened with two types of CFRP materials carbon fibre fabric (CFF) and silicone coated carbon fibre laminates (SCCFL), under static cyclic loading (load-controlled). Three series of beam specimens were static cyclically loaded until significant damage including unstrengthened control beams and beams strengthened with single and double layers of CFF and SCCFL. As key parameters ultimate load capacity, the load deflection behaviour, the energy dissipation, crack propagation and stiffness degradation were evaluated. Results indicated that strengthening of the beam with CFRP increased the nominal flexural strength, and its fatigue resistance. The two materials used for strengthening exhibited different performance behaviour, among which the SCCFL strengthened beams exhibited higher load capacity and energy dissipation, less crack width and retained stiffness under static cyclic loading (load-controlled). SCCFL is proved to be more effective in retrofitting RC structure to dynamic or fatigue loadings.

## Introduction

Reinforced concrete (RC) structures contributed a significant part to the built environment and are crucial parts of infrastructure systems including bridges, buildings, and industrial facilities. These constructions are susceptible to a number of types of degradation over time, such as corrosion of the steel reinforcement, excessive loading, and poor design considerations^1^. Structural post-strengthening operations are needed to meet specified service conditions. The externally bonded CFRP sheets are a promising element for structural rehabilitation works^2^. The benefits of CFRPs include a high strength-to-weight ratio, corrosion resistance, ease of use, and compatibility with existing structures. Its potential to reduce cracking, increased load carrying capacity, and extend the service life of deteriorated or underdesigned elements has drawn a lot of attention as one of its possible uses to increase the flexural capacity of RC beams^3,4^. The strength of the bond between the CFRP sheet and the concrete determines the structural behaviour of the reinforced concrete elements. The deterioration of the members could be greatly enhanced by CFRP strengthening in flexural and shear capacity as well as in extending their useful lives. Many structures are subjected to repeated load that the structure fails at a load lower than its static capacity^5–7^. Consequently, when rehabilitating concrete structures, fatigue loads or repeated loads should be considered. Experimental results demonstrated enhanced fatigue performance of RC beams with the use of external fiber-reinforced polymer strengthening, resulting in a 40% increase in service load capacity^8,9^. Additionally, CFRP has high fatigue strength under repeated loads^10^. Various research results showed that strengthening with external CFRP sheets in the tensile region of the RC beam considerably increases the strength at bending and reduces deflection, crack width, and load-bearing capacity of the member^11,12^. This method of strengthening the RC structures changes their behaviour under load and failure patterns^13^. Composite materials, such as fiber-reinforced polymers, are typically made from rigid, high-strength fibres like carbon, aramide, glass, or high-strength polyethylene that are embedded in epoxy resin. CFRP is a high-strength composite material made of carbon fibres wound around an epoxy resin matrix. CFRP strip strength is approximately 3000 MPa and comes in a thickness of up to 1.5 mm and a width up to 150 mm^14,15^. CFRP is a composite of carbon fibers embedded in an epoxy resin, with remarkable fatigue behavior and strength and stiffness in the fiber direction, and this is one of the factors that makes CFRP more resistant to corrosion than more traditional materials^16^. Composed primarily of carbon atoms, carbon is a material made up of incredibly thin fibres that range in diameter from 5 to 10 µm. Microscopic crystals that are roughly parallel to the fiber’s long axis are formed by the bonding of carbon atoms. The fibre is exceptionally strong for its size because of the crystal alignment. A yarn, which can be used alone or woven into fabric, is created by twisting several thousand carbon fibres together. First created as a sealant or bonding agent, silicone is a synthetic polymer that is both chemically inert and thermally stable. However, the use of silicone to coat carbon fibre fabric has increased due to the fabric market’s rapid expansion.

When utilizing the technique of near-surface mounted CFRP laminates in flexural faces, it was observed that the ultimate load of the beams increased by 21.74% and the stiffness by 14% in comparison to control beams^17–19^. External flexural strengthening by CFRP sheets shows enhanced performance in the shear capacity of the beams^19,20^. Increasing the weight of carbon fibre per unit area improved the beam’s strength capacity, and it could be utilized in place of several sheet layers^21^. Most of the research in field of strengthening with CFRP has been limited to monotonic or static loadings. However, in practice, the structures are subjected to a changing of a dynamic and repetitive characteristic of seismic activities, wind induced vibrations and traffic. Cyclic loading different from monotonic loading can lead to cumulative damage and a performance degradation. Therefore, the behaviour of CFRP reinforced RC beams subjected to cyclic loading has to be investigated for the reliability as well as the effectivity of the strengthening systems in practical applications. Cyclic loading subject the structural atmospheres to very different requirements than monotonic loading. Although monotonic tests offer some data on high load and the ultimate strength, cyclic loading inflicts cumulative damage, crack propagation and progressive stiffness failure and energy dissipation effects that are not reflected in the monotonic tests. Instructive examples of structures that are commonly subject to variability or repeated loads and where fatigue rules the long-term performance are those structures in bridges, in buildings in seismic zones, and road pavements under traffic loads. As such, the conduct of CFRP-strengthened RC beams under cyclic load is important not only to measure their strength but more important their durability, ductility and resilience to repeated stress states. Such a specific loading process requires methodical laboratory research so that the reinforcing techniques are not lost over the lifetime of the construction. Most of the research works have proved that CFRP sheets are effective in enhancing both flexural and fatigue performance of RC beams, but much research is still concentrated on either conventional carbon fibre fabrics or monotonic static loading case. There is a paucity of studies comparing the various CFRP configurations under repeated cyclic loading. The lack of direct comparative research between the uncoated carbon fibre fabric (CFF) loading and the silicone-coated carbon fibre laminates (SCCFL) loading under cyclic loading patterns inhibits the direct system comparison in terms of energy dissipation ability, stiffness retention and crack control in terms of their relative efficiencies. In light of filling this gap, the study presents a direct comparison of the load-carrying capacity of beams reinforced with CFF and SCCFL to help offer more definitive advice during the current retrofitting of RC structures exposed to fatigue or dynamic forces. The purpose of the study was to advance engineering understanding of flexural strengthening of RC beams using CFRP sheets from a cyclic loading perspective. Research is conducted by subjecting CFRP-strengthened beams to cyclic loading and assessing their performance in terms of failure mechanisms, stiffness degradation, crack propagation, and load redistribution. The fields of structural rehabilitation and retrofitting may benefit from these studies. The behaviour of CFRP strengthened RC beams under cyclic loading conditions was confirmed, providing insight into the planning and execution of a retrofitting strategy for the structures subjected to dynamic loading.

## Experimental program

In the experimental stage of the study, RC beams reinforced with CFF and SCCFL are being tested under cyclic loading in comparison to control beams. Three control beams and twelve beams with various CFRP alignments and strengthening techniques were subjected to flexural loads during testing. The primary factors surveyed were the number of CFRP layers, which ranged from one to two layers, and the fabric type of CFRP material, which included CFF and SCCFL sheets.

### Materials

**Fig. 1:**
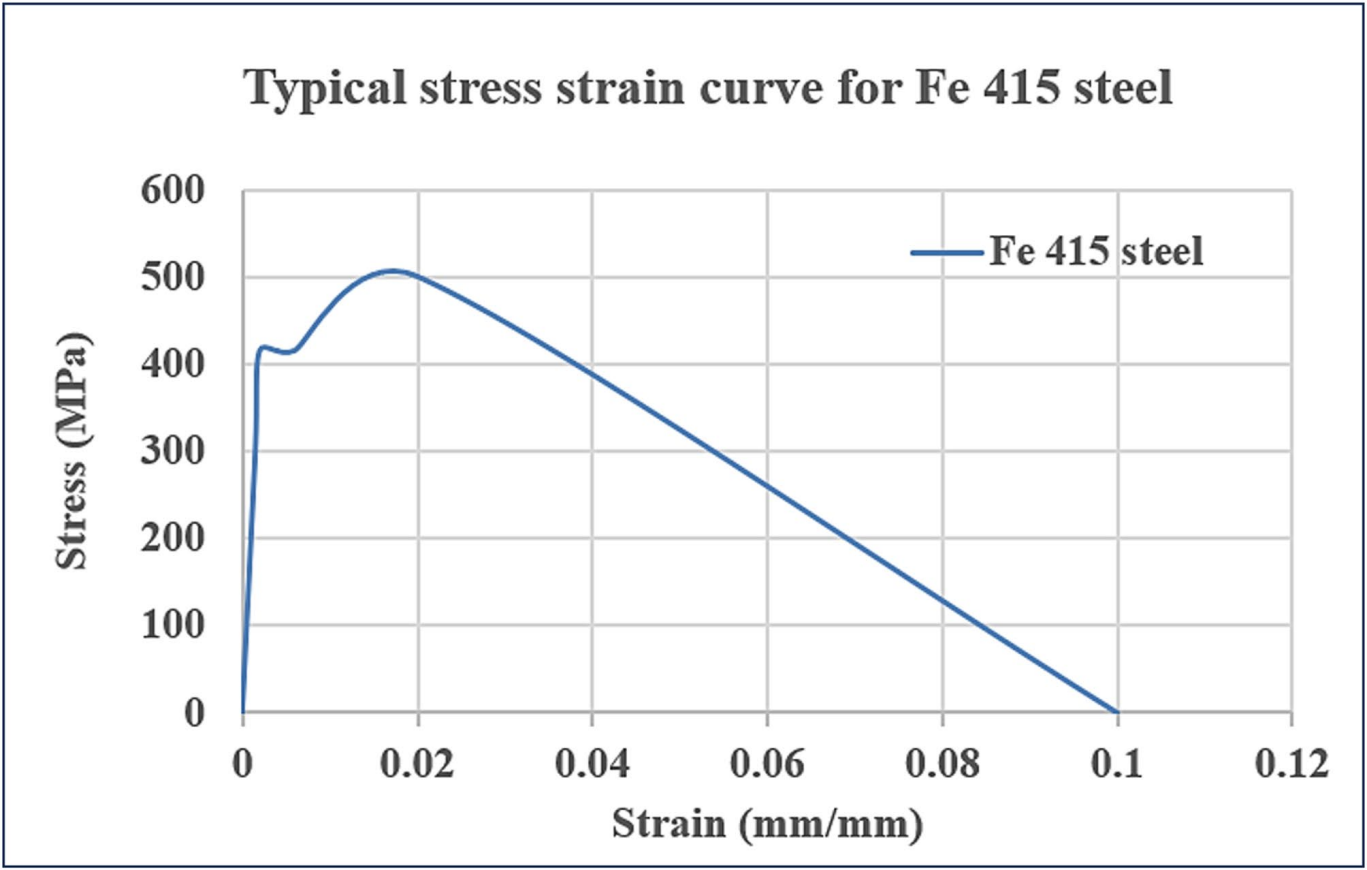
Typical stress–strain curve for Fe-415 steel.

**Fig. 2:**
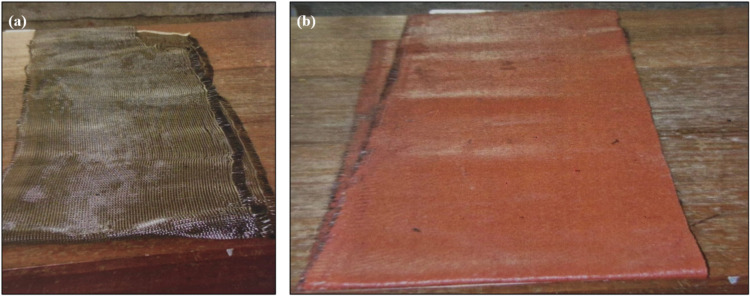
Images of (**a**) carbon fiber fabric, (**b**) silicone coated carbon fiber fabric.

Ordinary Portland cement (53 grade) confirming IS:12269–1987 is used in the concrete works throughout. Locally available crushed granite aggregates passing through a 20-mm sieve and retained on a 12.5-mm sieve with a fineness modulus of 7.9, confirming IS 383–1980, were used in the test procedure. Locally available natural river sand passing through a 4.75-mm-size sieve and retained on a 0.075-mm-size sieve with a fineness modulus of 2.7, confirming IS 383–1970, was used in the work. The steel of 415-grade tor steel was used for reinforcements following the specifications of IS 1786:2008 and IS 456:2000. Table 1 gives the details of the steel used for reinforcements and Fig. 1 gives the typical stress–strain curve for Fe-415 steel. Water of good quality was used throughout the work. With a target strength of 30 MPa, a normal-weight concrete mix design was employed. According to IS 456–2000, the concrete mix design had a water-to-cement ratio of 0.39 and cement, sand, and coarse aggregate ratios of 1:1.2:2.5. In this experimental study, an epoxy resin araldite GY 257 of specific gravity 1.8, a flexural strength of 450 to 550 kg/cm^2^, and a hardener HY 840 of specific gravity 2, a flexural strength of 300 to 400 kg/cm^2^, are mixed well in the ratio of 1.5:0.5 to have good adhesive and flowable properties and are used for pasting the CFRP sheets to the RC beams. Commercially available CFF from Chennai, India, with area weight of 204 GSM, fibre counts 5/cm, approximate dry thickness of 37 mm, tensile strength 830 MPa, young’s modulus 59 GPa, compression strength 460 MPa and interlaminar strength 61 MPa is used for the study. The fibres are distributed in the unidirectional longitudinal direction with a plain-weave, thus ensuring uniform stresses would be applied under flexural loading direction. Commercially available SCCFL from Chennai, India, with area weight of 600 GSM, fibre counts 37/cm, approximate dry thickness of 100 mm, tensile strength 570 MPa, young’s modulus 52 GPa, compression strength 420 MPa and interlaminar strength 45 MPa, as specified by the supplier’s datasheet, is used for the study. Figure 2 shows the images of carbon fiber fabric and silicone coated carbon fiber fabric respectively. The laminates are made in a uni-oriented fibre architecture and the coating of silicone on laminates has an average thickness of 0.25 mm uniformly on the surface to enhance layer adherence and environmental resistance. The area weight of the CFF used in this study is different from the SCCFL with 600, therefore, the two materials were purposely selected to represent different strengthening approaches, as thick woven carbon fibre fabric and a lightweight laminate with coating of silicone. These choices allow the direct comparison of the influence of fibre content, surface modification and bonding properties on the cyclic behaviour of the strengthened RC beams.

**Table 1:** Properties of steel reinforcement.

Property (Fe-415)	Values (Fe-415)
Yield strength (fy)	415 MPa (characteristic)
Ultimate tensile strength (fu)	485–600 MPa
Elongation (minimum)	14.5%
Modulus of elasticity (Es)	2.0 × 10^5^ MPa
Poisson’s ratio	0.3

### Concrete properties

Experimental methods were used to determine the characteristics of the concrete used in the study. In order to ascertain the compressive strength of concrete, nine specimens measuring 150 mm by 150 mm by 150 mm were cast in accordance with IS:456–2000 and exposed to the same curing conditions and beam element composition properties to guarantee a comparable compressive strength to that of the natural one. A compressive testing machine was used to test the cubes, and the average test result for all of the cubes was roughly 39.5N/mm^2^^22,23^. Table 2 provided specifics of the concrete strength test results after 28 days.

**Table 2:** Test results of concrete cubes.

S.No	Mix ratio	Failure load (tonnes)	Compressive strength (N/mm^2^)	Average compressive strength(N/mm^2^)
1	1: 1.2: 2.5, w/c = 0.39. (Cement: Sand: Coarse Aggregate)	106	37.11	38.29
2		111	39.33	
3		109	38.44	
4		118	42.44	39.74
5		110	38.89	
6		110	38.89	
7		113	40.22	40.52
8		121	43.78	
9		107	37.56	
Overall average compressive strength at 28 days = 39.5 N/mm2				

### Preparation of test beams

**Fig. 3:**
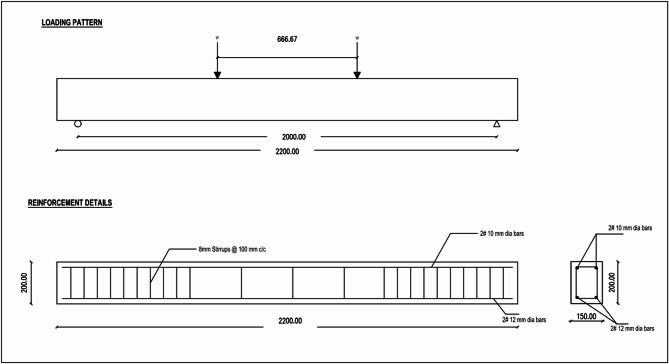
Typical beam cross-section details.

Fifteen RC beams measuring 2200 mm in length and 150 × 200 mm in cross-section were examined in this study. The beams were tested under a four-point bending setup, with a clear span of 1000 mm and a shear span-to-depth (a/d) ratio of 1.78, to evaluate their flexural performance. Two bars 10 mm in diameter were positioned at the compression zone and two bars 12 mm in diameter were positioned at the bottom of each beam as tensile reinforcement to hold the shear reinforcement in place during testing. 8-mm diameter stirrups were used to represent shear reinforcement^24–26^. They were spaced 100 mm apart at the midspan and 75 mm apart as they moved in the direction of the supports. Around the beam section, a 25 mm clear concrete cover was maintained. The flexural and shear reinforcements’ average steel yield strength was 415 MPa. The specifics of the beams used in this investigation are displayed in the table. The beams were divided into five groups. Group A consisted of the control beam specimens designated as C1, C2, and C3, that were not strengthened. Three beams in Group B, designated CF11, CF12, and CF13, were reinforced by a single layer of CFF. Three beams in Group C, designated CF21, CF22, and CF23, were reinforced with two layers of CFF. Group D contained three beams strengthened with one layer of SCCFL with names respectively as CS11, CS12, CS13. Group E contained three beams strengthened with two layers of SCCFL with respectively as CS21, CS22, CS23. The CFRP sheets were adhered to the concrete surface along the tension face of the beams for all of those strengthening projects using epoxy resin. Details and typical beam cross-sections are shown in the Fig. 3. With a clear span of 2000 mm and a total length of 2200 mm, the beam is supported by a hinge or fixed support at one end and a roller support at the other. The beam is subjected to symmetrical two-point loads of magnitude W, separated by 666.67 mm. The flexural performance of the beam under concentrated loads is frequently examined using this loading configuration. The cross-section of the beam is rectangular and measures 150 mm in width and 200 mm in depth. The two 10 mm diameter bars at the top and the two 12 mm diameter bars at the bottom make up longitudinal reinforcement; the latter is crucial for withstanding tensile stresses brought on by bending. Throughout the length of the beam, 8 mm diameter stirrups are positioned 100 mm center-to-center to improve shear resistance. The design ensures sufficient strength against shear forces and bending moments by adhering to IS: 456–2000’s standard reinforced concrete beam principles. The specimens were cast using steel moulds. A reinforcing cage was fabricated and placed inside the mould. The required quantities of cement, sand, and coarse aggregate are thoroughly mixed in a drum-type mixing machine. Water is added, and mixing is done for 10 min. Concrete is properly placed in the moulds and compacted using a table vibrator. After 24 h, the specimens were de-moulded and properly cured for 28 days.

### Strengthening of beams

**Fig. 4:**
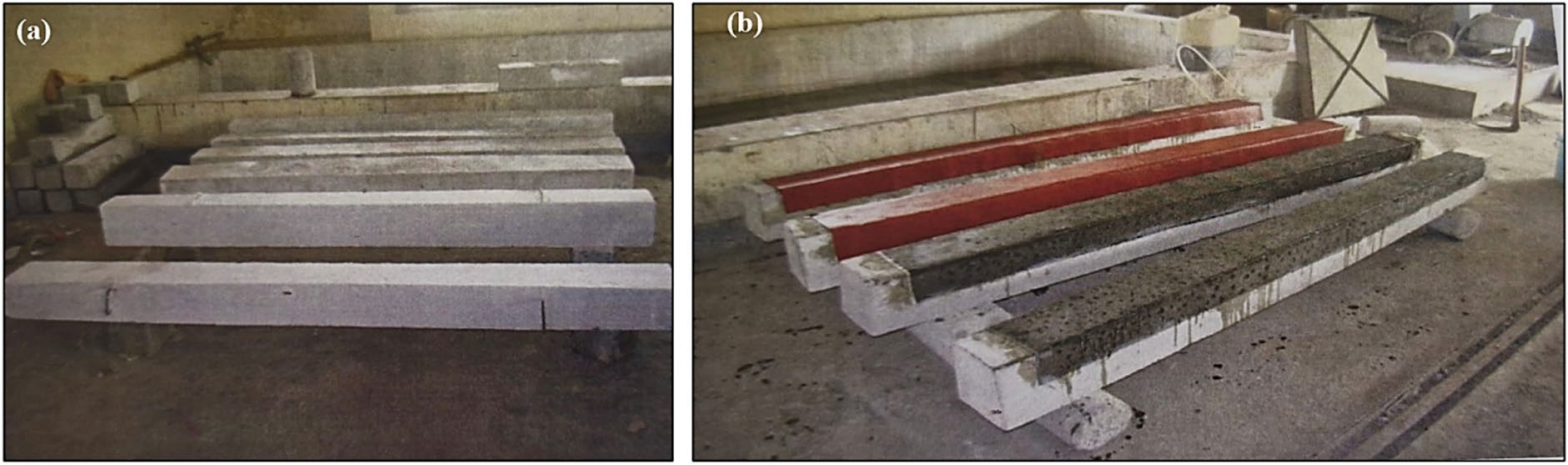
Images of beams (**a**) Control beams (**b**) strengthened beams during experimental program.

The same properties of reinforced concrete are used in the casting of each beam. After applying a coarse sandpaper texture to the concrete beams bottom flexural side, all dirt and debris were removed using an air blower. Beams named C1, C2, and C3 are used as the control beams with no strengthening. An average of three values was taken. Beams named CF11, CF12, CF13, CF21, CF22, and CF23 are those strengthened with CFRP fabric along the flexure face in one layer and two layers, respectively. Averages of three values were considered. Beams named CS11, CS12, CS13, CS21, CS22, and CS23 are those beams strengthened with SCCFL in one layer and two layers, respectively. An average of three values was taken^27–29^. For strengthening the beams, the surface of the beam is roughened by chipping and rubbing and then cleaned well. For the study, the epoxy resin and the hardener is mixed well in the ratio of 1.5:0.5 to have good adhesive and flowable property and is used for pasting the CFRP sheets to RC beams. A coat of epoxy resin mixed with hardener is applied uniformly over the surface. Then the CFRP sheets were laid over it uniformly in the proper dimensions. A further coating of epoxy resin mixed with hardener was applied over the fibre for proper bonding. Figure 4 shows the images of unstrengthened beams and strengthened beams during experimental the program.

### Test procedure

**Fig. 5:**
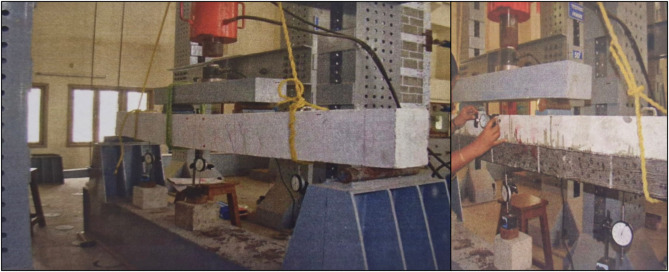
Test set up images during experimental program.

Figure 5 shows the test set up images during the experimental program. The loads were applied as two-point static cyclic loading at the middle third position of the test beams, which were simply supported over an effective span of 2000 mm. In increments of 0.5 kN, the load is gradually increased from zero and then decreased to zero. A dial gauge is used to measure the deflection at the midspan and the deflection at a third distance from both ends at each loading and unloading stage. A mechanical strain gauge is used to measure the strains at the tension and compression faces. A hydraulic jack was used to load each test beam. Strain gauges were attached to the beam tensile face at the midspan and close to the loading locations. The difference in the strain readings on the surface at every increment of the load was taken as an indicator to show the occurrence and spread of cracks. It was visually observed that cracks formed and expanded and compared to the strain measurement readings on each load stage. This cyclic loading was repeated until significant cracks and loss in strength were experienced.

A two-point loading scheme was adopted with reference to IS 516:2018 to allow a constant moment field in the central third of the beam that reduces shear effects and allows the direct measurement of flexural behavior characteristics including load capacity, ductility, and stiffness degradation. Testing of the beams was based on a 2-point static cyclic loading using a hydraulic jack. The increase and reduction of the load was done in steps of 0.5 kN and was initiated at zero, usually built up to the desired value, before reducing back to zero, and then repeating in each cycle. This happened until large cracking and the strength reduction was observed. The cyclic loading proceeded until failure, which was taken as one of the following as attainment of ultimate load capacity, attainment of midspan deflection of L/50 at 40 mm in the present beam span, occurrence of severe concrete crushing or wide flexural cracking, or debonding or rupture of CFRP, with a subsequent load drop of 20 or more percent. These stopping criteria provided strength and serviceability limit stops in repeated loading tests. Such a loading–unloading sequence was carried out until there was significant cracking and stiffness loss. The increment size was determined to ensure close observation of deflection and crack advancement and the target maximum load was set near the ultimate strength of control beams as used in prior research on CFRP-strengthened RC beams under cyclic loading cycles. All the experimental data which are reported in this study are described as an average of three replicated samples under each test condition. To measure the variability in the measured data, standard deviation (SD) was determined by the formula,

1 $$SD = \sqrt {\frac{{\begin{array}{*{20}c} \sum \\ {\sum {\left( {x_{i} - \overline{x}} \right)^{2} } } \\ \end{array} }}{n - 1}}$$

and the mean of the test values is a statistic for characterizing the degree of alienation. The statistical plot gives an indication of the reliability and repeatability of test findings^30–33^.

## Results and discussion

### Ultimate load-carrying capacities of beams

According to test results, the ultimate load supported by CFRP-sheet-strengthened beams was higher than that of control beams without strengthening, and the SCCFL-strengthened beams were higher than the CFF-strengthened beams. Similarly, for both CFF and SCCFL, the ultimate load supported by beams reinforced with double layers of CFRP sheets was higher than that supported by beams strengthened with single layers.

The Table 3 presents the test results of different RC beams, focusing on their ultimate load, deflection at failure, and flexural strength. Four groups of the beams are defined as control beams, beams with CFF reinforced, and beams reinforced with SCCFL. This helps comparing the impact of various approaches in the reinforcement of structural performance. For each beam, the values recorded pertain to its load bearing capacity and resistance to bending. Maximum force the beam withstands without failure is called ultimate load. Control beams exhibited the lowest average ultimate load of 38.66 kN in comparison with the strengthened beams. Results of CFF strengthened beams with one layer, exhibited improved performance with an average ultimate load of 50.07 kN, implying that the reason for structural capacity enhancement by fiber reinforcement. This capacity is further increased to 56.03 kN in the same beams reinforced with CFF but two layers of durable, which is shown to be more effective. Ranges of ultimate load values for the SCCFL reinforced beams had a range average of 63.73 kN and 77.9 kN. Features of the trend point out that strengthening layers make all the difference to the load bearing capabilities of the beams.

**Table 3:** Test results of beams.

Beams	Ultimate Load (kN)	Average Ultimate Load (kN)	S.D (kN)	Deflection at Ultimate Load (mm)	Average deflection (mm)	S.D (mm)	Moment Capacity (kNm)	Average Moment Capacity (kNm)	S.D (kNm)
C1	37.8	38.66	1.13	4.1	3.97	0.12	18.90	19.32	0.57
C2	38.2			3.9			19.10		
C3	39.9			3.9			19.95		
CF11	48.5	50.07	1.56	3.17	3.27	0.15	24.25	25.33	0.78
CF12	51.6			3.45			25.80		
CF13	50.01			3.2			25.00		
CF21	54.6	56.03	1.31	3.2	3.08	0.14	27.30	28.03	0.65
CF22	57.2			3.1			28.60		
CF23	56.3			2.93			28.15		
CS11	64.8	63.73	1.15	2.8	2.72	0.07	32.40	31.87	0.58
CS12	63.9			2.67			31.95		
CS13	62.5			2.7			31.25		
CS21	78.6	77.9	0.7	2.1	2.15	0.28	39.30	38.95	0.35
CS22	77.2			1.9			38.60		
CS23	77.9			2.45			38.95		

The flexural strength is a main measure of a beams bending. Results demonstrated the weakest structural performance correspond to the control beams with average flexural strength of 12.88 N/mm^2^. The addition of fiber reinforcement increased this property up to 17.24 N/mm^2^ for the CFF single layered beams and 20. 54 N/mm^2^ for the CFF double layered beams. In the SCCFL reinforced beams, the highest flexural strengths are obtained at 23.37 N/mm^2^ for single layer and 28.56 N/mm^2^ for double layer strengthened beams. This substantial improvement confirmed that additional layers significantly enhanced resistance to bending forces, making the SCCFL reinforcement the most robust among the tested beams.

### Load–deflection and energy dissipation behavior

**Fig. 6:**
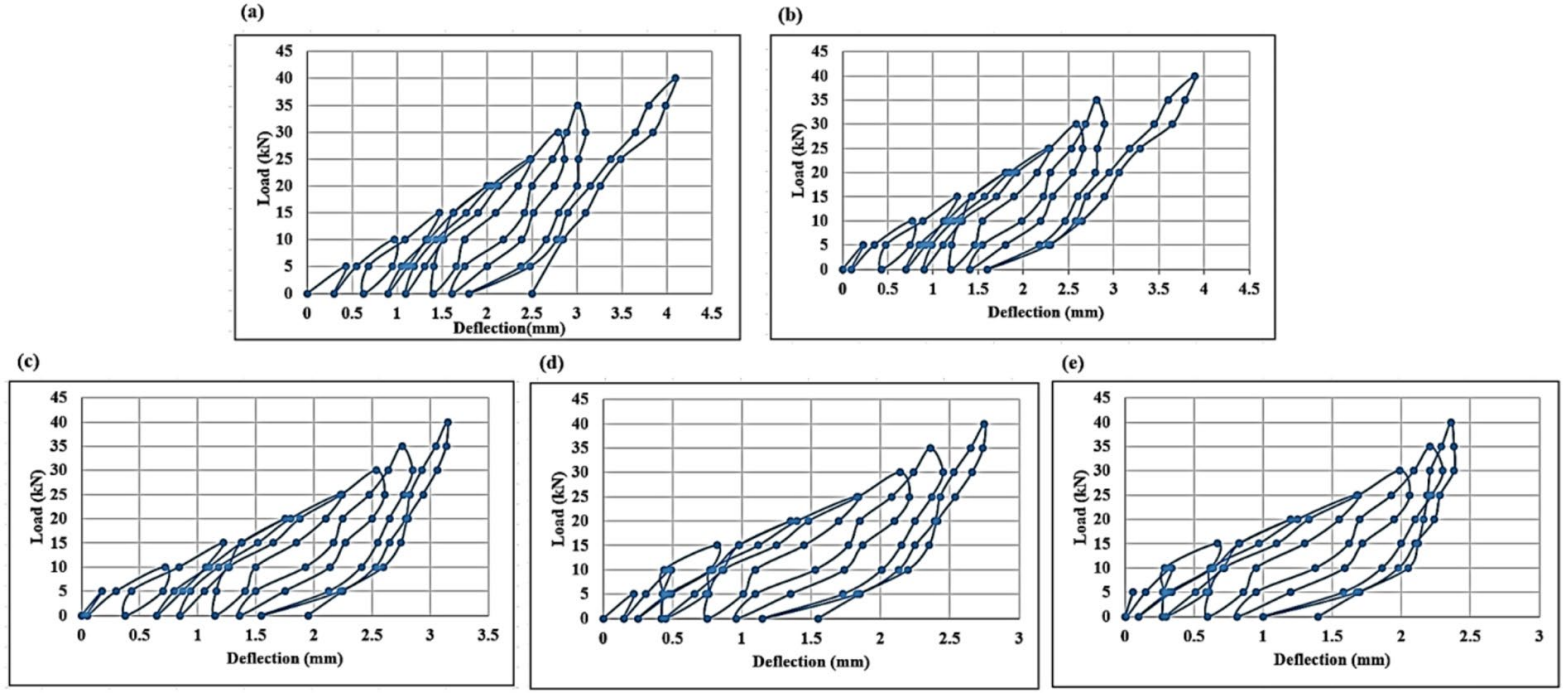
Load deflection curves for (**a**) control beams, (**b**) beams strengthened with 1-layer CFF, (**c**) beams strengthened with 2 layers of CFF, (**d**) beams strengthened with 1-layer SCCFL, (**e**) beams strengthened with 2 layers of SCCL.

The images in Fig. 6 shows the load deflection curves for the different beam specimens subjected to cyclic loading. The deflection in millimeters is on x axis with the load in kN on y axis. The plots show that there are hysteresis loops characteristic of cyclic tests. These loops indicate energy dissipation that may be through inelastic deformation, cracking, or internal damage mechanisms of the material. Insights into how durable and fatigue resistant the material is, comes from how it can endure repeated loading without immediate failure. Different strengthening techniques affect the mechanical response of each beam and each subplot corresponds to a different type of beam. Figure 6a depicts the typical load deflection response and no additional strengthening of the control beams. These beams were first used as a baseline of stiffness and deformation characteristics when loaded. Figure 6b was for beams strengthened with one layer of CFF. It is likely that the additional reinforcement helped the beam to carry more load, and resist excessive deformation. Thus, the extent of improvement is dependent on the bond strength and interaction between the CFF and concrete surface. The behavior of beams with two layers of CFF is illustrated for Fig. 6c. These beams are likely stiffer and better load resistant than Fig. 6b owing to the extra reinforcement. Figure 6d shows hysteresis loops that indicate that beams reinforced by one layer of the SCCFL resistant to damage progression and improve the energy dissipation than those involving solid, continuous concrete core. CFF would be stronger and possibly even better crack resistant, however if material properties and level of bonding is sufficient. This suggests that it has a better ductility and energy absorption as a function of load deflection behavior. The Fig. 6e is obtained by placing two SCCFL layers in beams. These beams are expected to be the ones with the highest capacity to withstand the load and the lowest deflection from the other specimens that have been tested. The beam is illustrated to be less prone to failure when loaded on subsequent loading due to its multiple hysteresis loops due to strengthening. Results show that CFF and SCCFL structural resilience with additional layers give a considerably higher increase in load capacity and reduced deflection.

Deflection at ultimate load indicates how much the beams bend before failure. The control beams show the highest deflection, ranging from 3.9 mm to 4.1 mm, implying that they experience more deformation under load. The fiber-reinforced CF1 and CF2 beams have slightly lower deflections of around 3.17 mm to 3.2 mm, suggesting increased stiffness. The CS-series beams display the lowest deflection values, with CS11-CS13 ranging from 2.67 mm to 2.8 mm and CS21-CS23 reaching as low as 1.9 mm to 2.45 mm. These results indicate that the beams reinforced with SCCFL significantly improved the beam rigidity, reducing deformation under stress.

**Fig. 7:**
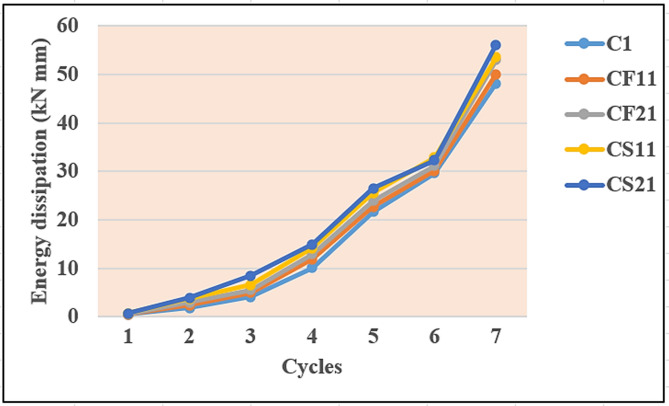
Energy dissipation curves.

One of the key considerations when assessing how well a reinforced concrete structure or structural element performs under extreme seismic activity is its capacity to dissipate the inelastic deformation energy. The area under the hysteresis loop, which begins and ends at the zero deflection points, is used in this study to define the energy dissipated in each loading cycle. The deflections of the beams for all load cycles at every increment of 0.5 tonnes were measured using mechanical strain gauges. A graph drawn between hysteresis loops of load and deflection indicates that the beams bonded with SCCFL show more energy dissipation than other beams. The energy dissipation for beam C1 during the first load cycle was 0.43 kNmm, and during the last cycle it was 49.13 kNmm. On average, the amount of energy dissipated for beams CF1, CF2, and CF3 was 0.51 kNmm during the first cycle and 52 kNmm for the last cycle, which indicates that the performance of beams strengthened with carbon fibre fabric was better compared with control beams with no strengthening. Similarly, the amount of energy dissipated by beams strengthened with SCCFL was 0.68 kNmm during the first cycle and 57.87 kNmm for the last cycle, indicating that the beams strengthened with SCCFL exhibited better performance when compared with control beams and beams strengthened with carbon fibre fabric. The calculated energy dissipation of the beams for various load cycles is presented in Fig. 7. The amount of energy dissipation is shown in the Table 4.

**Table 4:** Energy Dissipation capacities (kNmm).

Cycles	1	2	3	4	5	6	7
C1	0.43	1.8	4.05	10.1	21.63	29.6	48.13
C2	.45	1.9	4.1	10.5	21.9	29.9	48.5
C3	.43	2	4.2	10.7	22	30	48.9
CF11	0.48	2.4	4.89	11.1	22.13	30.1	49.99
CF12	0.47	2.5	4.7	11.5	22.5	30.2	49.5
CF13	0.48	2.3	4.6	11.89	22.7	30	50
CF21	0.51	2.7	5.01	12.09	23.1	30.15	52
CF22	0.51	2.6	5.2	12.5	23.5	30.5	52.5
CF23	0.50	2.6	5.3	12.7	23.9	30.7	50
CS11	0.55	2.7	5.5	13.1	24.5	31.87	52.58
CS12	0.56	2.7	5.5	13.5	24.7	31.2	53
CS13	0.57	2.8	5.7	13.7	25	32	52
CS21	0.6	2.9	7.43	13.9	25.5	32.25	55.13
CS22	0.62	2.8	6.5	14.1	25	32.5	54
CS23	0.68	3.7	6.2	14.21	25.5	32.13	57.87

The graph illustrates the load–deflection behaviour of different types of concrete beams under flexural testing. The x-axis likely represents deflection in mm, while the y-axis represents the applied load in kN. Each curve corresponds to a different beam type, including the control beam C1, carbon fiber fiber-reinforced beams named CF11 and CF21, and SFCCL strengthened beams named CS11 and CS21. Initially, all beam types exhibit a similar trend, indicating comparable stiffness in the early stages of loading. As the load increases, differences in performance become more evident, with the SFCCL strengthened beams CS11 and CS21 and CFF reinforced beams CF11 and CF21 demonstrating higher load-carrying capacities compared to the control beam. The CS-series beams, in particular, show the highest ultimate load, suggesting that supplementary materials or advanced reinforcement techniques significantly improve structural strength. The curves indicate that reinforcement enhances both load resistance and stiffness, leading to reduced deflection at higher loads. Overall, the graph highlights the effectiveness of fiber reinforcement and additional strengthening methods in improving beam performance under flexural loading.

### Cracking behavior and stiffness degradation

At a load of 1.5 kN, the control beams showed the first signs of cracking. At a load of 3.5 kN, the first crack in beams CF11, CF12, and CF13 emerged. While the first crack in beams CS series appeared at loads greater than 4.5 kN, the first crack in beams CF series appeared at a load of 4.2 kN. When comparing RC beams to control beams and beams reinforced with carbon fibre fabric under static cyclic loading, the width of the cracks was also observed to be smaller than in control beams, suggesting that SCCFL is a superior flexural strengthening technique. Upon loading, it was discovered that the reinforced beam specimens were more robust and rigid than the control specimens. It was observed that the control beams’ stiffness varied from 19.23 kN/mm in the first loading cycle to 11.11 kN/mm in the final loading cycle. Comparatively, the stiffness values of strengthened were found to be reduced, indicating they were ductile. There was a general degradation of stiffness concerning an increase in load cycles. The initial observable flexural fissure was observed in the control beams at mid-span with load reaching about 1.5 kN. Progressive deflection by cyclic loading caused these cracks to expand and vertically stretch towards the compression zone, which later crushed the top fibre of concrete towards the mid-span. Cracks started later at about 3.5kN in beams reinforced by a single layer of CFF (CF11, CF12, CF13). The cracks were smaller than the control beams and were spaced throughout the span, although at increased cycles of loading, debonding taking place both between the CFF sheet and the concrete surface was noted and thus, this became the characteristic mode of failure. In two-layered (CF21, CF22, CF23beams), the crack initiation was further postponed at about 4.2 kN. These beams had several small cracks with smaller widths and failure was caused by progressive debonding of the fibre sheets instead of sudden tearing. In beams reinforced with SCCFL (CS11, CS12, CS13 beams), the cracking initiation was greatly prolonged with the initial crack emerging at its loads exceeding 4.5 kN. Cracks were smaller and evenly spread in comparison to both control and CFF-strengthened beams. In the case of beams with two layers of SCCFL (CS21, CS22, CS23 beams), the crack pattern was well controlled and failure was not sudden and unforeseen but was gradual, with stiffness reduction. The SCCFL layers were well bonded with local debonding observed as failure approached. These physical observations validated that SCCFL reinforcement provided better crack control and more ductile failure behaviour over CFF and control beams.

### Failure modes

The failure modes of the beams depended on the kind of strengthening. Control beams collapsed due to large-scale flexural cracking, which was followed by the crushing of the concrete in compression. CFF-strengthened beams were partially debonded at interfaces between the fabric and concrete substrate before ultimate failure and showed localized crushing of the concrete. Conversely, the end-effected beams exhibited little interfacial debonding. Bonding strength offered by the silicone coating retarded bond failure, and bond failure was typically marked by concrete crushing in the compression area, and in other instances, tearing of the SCCFL sheet over repeated cycles. These failure modes are unique to SCCFL and they establish the high bond integrity and efficacy of SCCFL over CFF. The graphical observation of the specimens under test has shown that SCCFL-enforced beams had less and less drastic interfacial cracks in contrast to beams enhanced with CFF sheets only. This observation is associated with the enhanced load carrying capacity and energy dissipation characteristics of the beams made of SCCFL. Nonetheless, microscopic analysis was not done in the present study and further research will include microstructural examination that will support this behaviour.

## Conclusions

Through this experimental investigation, it was shown that the use of CFRP sheets in reinforced concrete beams under cyclic loading is quite effective. The increase in the ultimate loads for carbon fibre fabric (CFF) strengthened beams in respect to control beams was about 10.7% while that for silicone coated carbon fibre laminates (SCCFL) strengthened beams was about 26.86%. It was also observed that SCCFL strengthening resulted in later crack initiation, lower incremental stiffness degradation of beams per loading cycle and reduction at the fatigue resistance. The results indicated that SCCFL is particularly suitable to the rehabilitation of RC structures under repetitive or dynamic loading conditions, for instance, in seismic regions and urbanized places. The following conclusions are notable.

1. Studies showed that RC beams gained strength through the application of CFRP sheets for strengthening purposes.
2. The results show that the ultimate load carrying capacity of the beams strengthened with CFF increased by 10.7% and beams strengthened by SCCFL increased by 26.86% when compared with the ultimate load of control beams.
3. From the hysteresis loop of load vs deflection, it is observed that the beams strengthened with SCCFL gives higher energy dissipation and is more suited for structures subjected to cyclic loading.
4. The first crack appearance was at the load of 1.5 kN in control beams whereas in CFRP strengthened beams the first crack appeared at a load of 3.5 kN. Further the width of the cracks of strengthened beams are less compared to control beams.
5. While comparing the load bearing capacity and flexural behaviour of CFF and SCCFL strengthened beams, the SCCFL strengthened beams showed better performance.

## Data Availability

The data used to support the findings of this study are included in the article. Should further data or information be required, these are available from the corresponding author upon request.

## Abbreviations

RC: Reinforced concrete
CFRP: Carbon fibre reinforced polymer
CFF: Carbon fibre fabric
SCCFL: Silicone-coated carbon fibre laminates
